# Oscillometry and spirometry are not interchangeable when assessing the bronchodilator response in children and young adults born preterm

**DOI:** 10.1002/ppul.26632

**Published:** 2023-08-04

**Authors:** Elizabeth F. Smith, Tiffany K. Bradshaw, Rhea C. Urs, Denby J. Evans, Naomi R. Hemy, Graham L. Hall, Andrew C. Wilson, Shannon J. Simpson

**Affiliations:** ^1^ Wal‐Yan Respiratory Research Centre, Telethon Kids Institute Perth Children's Hospital Nedlands Australia; ^2^ Curtin School of Allied Health Faculty of Health Sciences Bentley Australia; ^3^ Child and Adolescent Health Service Perth Children's Hospital Nedlands Australia

**Keywords:** bronchodilator response, preterm, respiratory physiology

## Abstract

**Introduction:**

The European Respiratory Society Oscillometry Taskforce identified that clinical correlates of bronchodilator responses are needed to advance oscillometry in clinical practice. The understanding of bronchodilator‐induced oscillometry changes in preterm lung disease is poor. Here we describe a comparison of bronchodilator assessments performed using oscillometry and spirometry in a population born very preterm and explore the relationship between bronchodilator‐induced changes in respiratory function and clinical outcomes.

**Methods:**

Participants aged 6–23 born ≤32 (*N* = 288; 132 with bronchopulmonary dysplasia) and ≥37 weeks' gestation (*N* = 76, term‐born controls) performed spirometry and oscillometry. A significant bronchodilator response (BDR) to 400 μg salbutamol was classified according to published criteria.

**Results:**

A BDR was identified in 30.9% (*n* = 85) of preterm‐born individuals via spirometry and/or oscillometry, with poor agreement between spirometry and oscillometry definitions (*k* = 0.26; 95% confidence interval [CI] 0.18–0.40, *p* < .001). Those born preterm with a BDR by oscillometry but not spirometry had increased wheeze (33% vs. 11%, *p* = .010) and baseline resistance (Rrs_5_ z‐score mean difference (MD) = 0.86, 95% CI 0.07–1.65, *p* = .025), but similar baseline spirometry to the group without a BDR (forced expiratory volume in 1 s [FEV_1_] z‐score MD = −0.01, 95% CI −0.66 to 0.68, *p* > .999). Oscillometry was more feasible than spirometry (95% success rate vs. 85% (FEV_1_), 69% (forced vital capacity) success rate, *p* < .001), however being born preterm did not affect test feasibility.

**Conclusion:**

In the preterm population, oscillometry is a feasible and clinically useful supportive test to assess the airway response to inhaled salbutamol. Changes measured by oscillometry reflect related but distinct physiological changes to those measured by spirometry, and thus these tests should not be used interchangeably.

## 1 INTRODUCTION

The clinical review by members of the European Respiratory Society Oscillometry Taskforce^1^ identified that oscillometry may have a key role in the management of survivors of very preterm birth (delivered <32 weeks completed gestation).^2^ Over the lifespan, survivors of very preterm birth report increased respiratory symptoms, including wheeze, inhaled asthma medication use, and rehospitalization during early childhood compared with their term‐born counterparts.^3^ Lung function deficits, including reduced forced expiratory volume in 1 s (FEV_1_), and abnormal respiratory mechanics, are reported throughout childhood and into adulthood.^4^, ^5^, ^6^, ^7^ By school‐age, approximately 50% of very preterm‐born children are diagnosed with asthma^5^; up to five times increased odds than those born at term.^8^ Despite the high prevalence of asthma diagnoses in this patient group, preterm lung disease is typically non‐atopic^9^ with low exhaled nitric oxide (FeNO),^10^ contrary to childhood asthma. Additionally, recent trials of inhaled corticosteroids (ICS) report only modest improvements in lung function.^11^

Even with ICS therapy, a degree of airway reversibility exists for those born <32 weeks gestation.^11^ A significant bronchodilator response has been reported in about one‐third of those born preterm,^12^ with the highest rates in those with a neonatal diagnosis of chronic lung disease of prematurity, bronchopulmonary dysplasia (BPD). Studies report 25%–60% of school‐aged children with BPD respond to bronchodilators.^7^, ^12^, ^13^, ^14^ Despite this, there are reports of preterm‐born children being undertreated with bronchodilators, possibly due to the belief that respiratory symptoms in this group are an inevitable consequence of airway injury and remodeling.^15^ Further, recent findings from our group indicate that those most likely to respond to inhaled corticosteroids, display a degree of airway reversibility.^16^ A thorough assessment of the efficacy of short‐acting bronchodilators is likely to become key to optimal patient management in this group.

The response to inhaled bronchodilators is typically assessed using spirometry, however, the assessment of the bronchodilator response by oscillometry may offer additional advantages in the evaluation of preterm lung disease. As highlighted by the recent ERS review,^5^ there is evidence that oscillometry may be a useful tool in this patient group. At baseline, oscillometry outcomes are abnormal in those born very premature, with the worst abnormalities observed in those with BPD.^6^, ^17^, ^18^ Additionally, in those born <32 weeks gestation, oscillometry outcomes correlate with respiratory symptoms^5^, ^18^ and are sensitive to changes in lung function due to exposure to tobacco smoke.^19^ High test feasibility may be of particular value in this population where patients are young and developmental delay is associated with severe respiratory disease.^20^ Despite these advantages, the utility of oscillometry for the assessment of bronchodilator responses in preterm lung disease has yet to be explored.

Whilst few studies currently exist examining the bronchodilator response by oscillometry in those born preterm,^7^, ^18^ asthma studies have reported that an oscillometry assessment of the bronchodilator response may be better than spirometry at differentiating asthmatic from healthy children^21^, ^22^ and identifying individuals with poor asthma control.^23^ Emerging evidence suggests that intra‐breath oscillometry may identify a bronchodilator response in smokers and patients with COPD with greater sensitivity than spirometry.^24^ Due to its ability to detect changes in the small airways, it may be that oscillometry is a more sensitive test in assessment of the bronchodilator response in those born preterm, however, this has yet to be determined.

This study aimed to assess the feasibility and sensitivity of detecting a bronchodilator response by spirometry and oscillometry using published cut‐offs in a preterm population. To further our understanding of the interpretation of these tests, we aimed to investigate the correlations and agreement between reported outcomes, and their association with clinical symptoms. We hypothesized that a greater response to bronchodilators would be observed in those born preterm (by all methods). We further hypothesized that there would be a correlation between oscillometry and spirometry bronchodilator‐induced changes, but that oscillometry outcomes would correlate with symptoms and identify individuals with a bronchodilator response that would not have been identified by spirometry alone.

## 2 METHODS

### 2.1 Participants

Preterm‐born children and young adults, with and without a diagnosis of BPD, and healthy term‐born controls, were assessed between the ages of 6 and 23 years (data are collated from two distinct cohorts ages 6–12^16^ and 16–23^25^). Elements of this lung function data have been presented in these publications. Preterm‐born participants were delivered at 32 weeks gestation or less, hospitalized at King Edward Memorial Hospital (KEMH) in Perth, Western Australia. Participants born preterm were classified as having bronchopulmonary dysplasia if they received 28 days of oxygen supplementation or more, as assessed at 36 weeks postmenstrual age.^26^ Healthy term participants were born at 37 weeks gestation or more and had no history of recurrent respiratory symptoms or lung disease at the time of recruitment. Written informed consent was obtained from participants over 18 years of age and from parents or guardians of participants under 18 years. Ethical approval was obtained from the Child and Adolescent Health Service Human Research Ethics Committee (RGS367, RGS815).

### 2.2 History and symptoms

Neonatal and maternal health data was obtained from medical records and the KEMH neonatal database. Respiratory symptoms history was obtained using validated general and respiratory questionnaires adapted from the International Study of Asthma and Allergies in Childhood (ISAAC) questionnaires.^27^ Respiratory symptoms such as wheeze, shortness of breath, cough and rattly chest in the 3 months before the participant's study visit were parentally or self‐reported, as appropriate.

### 2.3 Lung function assessment

Participants attended Perth Children's Hospital for lung function assessment. Respiratory mechanics were assessed using the TremoFlo C‐100 (Thorasys Inc.). Spectral oscillometry (average impedance across the entire breathing cycle) was performed across frequencies of 5–37 Hz. Oscillometry was also performed using a single 10 Hz waveform to assess within‐breath impedance, allowing deconvolution of the separate phases of the breathing cycle and, therefore, inspiratory and expiratory impedance to be reported. Spirometry was performed using the Medisoft Hypair or BodyBox 5500 (Medisoft Corporation). All tests were carried out according to American Thoracic Society/European Respiratory Society (ATS/ERS) guidelines.^1^, ^28^, ^29^

Spirometry outcomes were expressed as z‐scores according to the Global Lung Function Initiative equations.^30^ Spectral oscillometry outcomes, including respiratory resistance at 5 Hz (Rrs_5),_ resonant frequency (Fres), area under the reactance curve (AX), and respiratory system reactance at 5 Hz (Xrs_5_) were expressed as z‐scores according to the reference equations published by Calogero et al.^31^ (6–12 year data) and Oostveen et al.,^32^ (16–23 year data). Rrs_5‐20_, and intra‐breath oscillometry measures were expressed as raw values and absolute difference.^33^

Oscillometry and spirometry were performed before and after administration of 400 µg salbutamol via a spacer. A bronchodilator response by spirometry was defined according to ATS/ERS guidelines as an increase of ≥200 mL and 12% in FEV_1_ or forced vital capacity (FVC)^29^ (the 200 mL rule was omitted for children ≤12 years). A bronchodilator response by spectral oscillometry was defined according to ERS guidelines as a change of ≤−40% in Rrs_5_, ≥50% in Xrs_5_, or ≤−80% in AX across all age groups.^1^

### 2.4 Statistics

Data was analyzed using IBM SPSS Statistics for Windows, Version 27.0 and GraphPad Prism 9.4.0. Normally distributed data are presented as means and standard deviations. Non‐normally distributed data are presented as medians and interquartile ranges. Differences between two groups were analyzed by independent samples *t*‐test or Mann–Whitney *U* test depending on normality of the data. To compare three or more groups, the one‐way analysis of variance with Bonferroni Post Hoc or the Kruskal–Wallis test with pairwise comparisons was performed, as appropriate. For categorical data, the chi‐squared test was used. Agreement between spirometric and oscillometric BDRs was assessed using kappa statistics, where Cohen's kappa coefficient (*k*) of >.75 represented excellent agreement, 0.40–0.75 represented a fair to good agreement, and <0.40 was indicative of poor agreement.^34^

## 3 RESULTS

### 3.1 Study participants

Included in the study were 364 participants (76 term; 288 preterm) at a median (IQR) age of 12.9 years (9.8–19.0), assessed at a single time point. There were no anthropometric differences between the preterm and term cohorts (Tables 1 and SE1). Those born ≤32 weeks gestation had a high burden of respiratory symptoms, with 39% having received an asthma diagnosis during their lifetime. Symptom burden was greatest in those with BPD (Table SE1).

**Table 1:** Participant demographics and respiratory symptoms.

	Term	Preterm	*p*‐Value
Number	76	288	
Neonatal characteristics			
BPD, *N* (%)		132 (45.8%)	
Gestational age, wks		28.14 (26.0–30.0)	
Birth weight, z‐score		−0.06 ± 0.86	
Supplemental oxygen support in NICU, days		8.3 (0.1–75.0)	
Total respiratory support in NICU, days		18.5 (2.9–55.0)	
Received postnatal surfactant, *N* (%)		205 (71.2%)	
Participant demographics			
Age, years	17.0 (9.6, 19.5)	12.9 (9.9, 18.8)	.721
Male, *N* (%)	38 (50.0%)	159 (55.2%)	.418
Height, cm	156.8 ± 22.6	152.1 ± 19.7	.099
Weight, kg	52.4 ± 22.8	48.6 ± 21.6	.195
BMI	20.1 ± 4.3	19.9 ± 4.8	.716
Asthma ever, *N* (%)	5/73 (6.8%)	109/279 (39.1%)	<.001*
Asthma medication – past 3 months, *N* (%)	2/73 (2.7%)	40/280 (14.3%)	.007*
In the past 3 months, when the participant did not have a cold, they have experienced:			
Wheezing, *N* (%)	4/73 (5.5%)	46/277 (16.6%)	.016*
Wheeze during exercise, *N* (%)	2/73 (2.7%)	44/277 (15.9%)	.003*
Coughing, *N* (%)	30/73 (41.1%)	141/278 (50.7%)	.143
A rattle in the chest, *N* (%)	9/73 (12.3%)	44/277 (15.9%)	.451
Shortness of breath, *N* (%)	10/73 (13.7%)	77/277 (27.8%)	.013*

*Note*: Total respiratory support includes nasal continuous positive airway pressure, humidified high flow, and mechanical ventilation. Participant demographics at the time of testing are presented as *n* (%), mean (SD), or median (IQR).

Abbreviations: BMI, body mass index; BPD, bronchopulmonary dysplasia; IQR, interquartile range; SD, standard deviation.

* *p* < .05 compared with the term‐born group.

### 3.2 Baseline lung function

Preterm participants had a lower FEV_1_ (MD = −0.87; 95% confidence interval [CI] −0.56 to −1.17; *p* < .001) and FEV_1_/FVC z‐score (MD = −0.85; 95% CI −0.57 to −1.14; *p* < .001), but not FVC z‐score (Table 2) compared with term‐born participants. Abnormal spirometry (defined as FEV_1,_ FVC, or FEV_1_/FVC ≤ − 1.64 z‐scores) was observed in 86/264 (32.6%) of preterm participants, compared with 7/70 (10%) of term born controls (*p* < .001), with the worst lung function seen in those with a neonatal diagnosis of BPD (Table SE2). Specifically, 47/264 (17.8%) preterm participants had abnormal FEV_1_ (2/70; 2.9% term), 69/217 (31.8%) preterm participants had abnormal FEV_1_/FVC (4/59; 6.8% term) and 7/217 (3.2%) preterm participants had abnormal FVC (3/59; 5.1% term).

**Table 2:** Baseline lung function in term and very preterm participants.

	Term	Preterm	*p*‐Value
Spirometry			
FEV_1_, *N* valid (%)	70 (92.1%)	264 (91.7%)	.902
FEV_1_ z‐score	0.26 ± 1.12	−0.61 ± 1.21	<.001*
FVC, *N* valid (%)	59 (77.6%)	217 (75.3%)	.679
FVC z‐score	0.40 ± 1.14	0.10 ± 1.06	.071
FEV_1_/FVC z‐score	−0.21 ± 0.93	−1.06 ± 1.07	<.001*
Spectral oscillometry			
Spectral, *N* valid (%)	72 (94.7%)	277 (96.2%)	.573
Rrs_5_ z‐score	0.35 ± 1.31	0.58 ± 1.29	.185
Xrs_5_ z‐score	−0.52 ± 0.90	−1.04 ± 1.29	<.001*
AX z‐score	0.82 ± 0.99	1.49 ± 1.30	<.001*
Fres z‐score	0.76 ± 1.05	1.38 ± 1.31	<.001*
Rrs_5‐20_ (cmH_2_O.s.L^−1^)	0.28 (0.01–0.82)	0.73 (0.17–1.63)	<.001*
Intrabreath oscillometry			
Intrabreath, *N* valid (%)	73 (98.6%)	268 (97.5%)	.542
R10_insp_ (kPa.s.L^−1^)	4.01 (2.70–5.07)	4.73 (3.37–6.58)	.002*
R10_exp_ (kPa.s.L^−1^)	4.43(2.97–5.64)	5.10 (3.48–6.93)	.008*
R10_insp‐exp_ (kPa.s.L^−1^)	−0.35 (−0.55 to −0.12)	−0.21 (−0.53 to 0.02)	.026*
X10_insp_ (kPa.s.L^−1^)	−0.65 (−1.22 to −0.23)	−1.08 (−1.94 to −0.54)	<.001*
X10_exp_ (kPa.s.L^−1^)	−0.77 (−1.51 to −0.29)	−1.32 (−2.44 to −0.61)	<.001*
X10_insp‐exp_ (kPa.s.L^−1^)	0.18 (0.00–0.47)	0.19 (−0.07 to 0.55)	.561

*Note*: Data are presented as mean ± SD or median (IQR), except for the number of valid tests.

Abbreviations: AX, area under the reactance curve; FEV_1_, forced expiratory volume in 1 s; Fres, resonant frequency; FVC, forced vital capacity; IQR, interquartile range; Rrs_5_, respiratory system resistance at 5 Hz; Rrs_5‐20_, difference in respiratory system resistance between 20 and 5 Hz; SD, standard deviation; Xrs_5_, respiratory system reactance at 5 Hz.

* *p* < .05 compared with the term‐born group.

Preterm‐born participants also had abnormal respiratory mechanics as assessed by oscillometry. Spectral oscillometry z‐scores revealed significant differences in reactance at 5 Hz (Xrs_5_) (MD = −0.52; 95% CI −0.26 to −0.79; *p* < .001), area under the reactance curve (AX) (MD = 0.67; 95% CI 0.40–0.94; *p* < .001) and resonant frequency (Fres) (MD = 0.62; 95% CI 0.33–0.91; *p* < .001) for those born preterm, compared to term. Z‐scores for respiratory resistance at 5 Hz (Rrs_5_) were not different between term and preterm groups (Table 2, *p* > .05), however the difference between respiratory resistance at 5 and 20 Hz (Rrs_5‐20_) was greater in the preterm group (Table 2, *p* < .001).

At 10 Hz, pre‐bronchodilator inspiratory and expiratory resistance was higher and reactance was lower in the preterm group (Table 2), however, the magnitude of the difference between inspiratory and expiratory reactance values (X10_insp‐exp_) was not different in those born preterm compared to term‐born controls (Table 2, *p* > .05).

### 3.3 Assessment of the bronchodilator response

Oscillometry was more feasible than spirometry, with 95% of overall participants obtaining acceptable measures pre‐ and postbronchodilator with oscillometry, compared to 85% for FEV_1_ (*p* < .001) and 68% for FVC (*p* < .001). However, the feasibility of achieving a successful bronchodilator assessment with either test was similar in term and preterm groups (*p* > .05, Table 3).

**Table 3:** Lung function changes in response to 400 μg salbutamol in term and preterm participants.

	Term	Preterm	*p*‐Value
Spirometry			
FEV_1_ pre & post‐BD, *N* valid (%)	66 (86.8%)	245 (85.1%)	.697
ΔFEV_1_, L	0.14 (0.10–0.18)	0.20 (0.17–0.22)	.016*
ΔFEV_1_, %	4.4 (3.2–5.5)	7.8 (6.9–8.7)	<.001*
FVC pre & post‐BD, *N* valid (%)	57 (75.0%)	195 (67.7%)	.221
ΔFVC, L	−0.01 (−0.04 to 0.01)	0.01 (−0.01 to 0.02)	.139
ΔFVC, %	−0.4 (−1.2 to 0.4)	0.4 (−0.1 to 0.9)	.108
Spectral oscillometry			
Pre & post‐BD, *N* Valid (%)	71 (93.4%)	275 (95.5%)	.460
ΔRrs_5_, %	−19.1 (−22.6 to −15.6)	−23.9 (−25.5 to −22.3)	.013*
ΔXrs_5_, %	13.2 (8.0–18.3)	24.6 (22.3–26.8)	<.001*
ΔAX, %	−30.0 (−37.7 to −22.3)	−44.1 (−47.7 to −40.6)	.001*
ΔRrs_5‐20,_ (kPa.s.L^−1^)	−0.14 (−0.27 to −0.01)	−0.54 (−0.64 to −0.43)	<.001*
Intrabreath oscillometry			
Pre & post‐BD, *N* Valid (%)	*71 (93.4%)*	*266 (92.4%)*	.754
ΔR10_insp,_ (kPa.s.L^−1^)	−0.16 (−0.45 to 0.12)	−0.49 (−0.71 to −0.27)	.074
ΔR10_exp,_ (kPa.s.L^−1^)	−0.14 (−0.42 to 0.14)	−0.38 (−0.61 to −0.16)	.175
ΔR10_insp‐exp,_ (kPa.s.L^−1^)	−0.02 (−0.16 to 0.11)	−0.11 (−0.18 to −0.03)	.305
ΔX10_insp,_ (kPa.s.L^−1^)	0.34 (0.24–0.45)	0.60 (0.48–0.72)	.002*
ΔX10_exp,_ (kPa.s.L^−1^)	0.22 (0.08–0.36)	0.58 (0.43–0.72)	<.001*
ΔX10_insp‐exp,_ (kPa.s.L^−1^)	0.03 (−0.06 to 0.12)	0.02 (−0.04 to 0.09)	.861

*Note*: Data are presented as mean difference (post‐pre bronchodilator values) or percent change (((post‐pre bronchodilator)/pre‐bronchodilator)×100) (95% CI). T‐Tests were used to identify differences between the term and preterm group.

Abbreviations: AX, area under the reactance curve; BD, bronchodilator; FEV_1_, forced expiratory volume in 1 s; FVC, forced vital capacity; Rrs_5_, respiratory system resistance at 5 Hz; Rrs_5‐20_, difference in respiratory system resistance between 20 and 5 Hz.

* *p* < .05.

A greater BDR was observed in the preterm group compared to the term group via both spirometry and oscillometry (Table 3). A small but significant improvement in FEV_1,_ but not FVC, was observed, relative to term‐born controls with a mean difference of 3.5% (95% CI 2.0–4.9; *p* < .001). Similarly, improvements were observed in the oscillometry measures ΔRrs_5_ (MD = −4.9%, 95% CI −8.7 to −1.0, *p* = .013), ΔRrs_5‐20_ (MD = −0.39, 95% CI −0.56 to −0.23, *p* < .001), ΔXrs_5_ (MD = 11.4%, 95% CI 5.8 to 17.0, *p* < .001) and ΔAX (MD = −14.1%, 95% CI −22.6 to −5.7, *p* = 0.001). The magnitude of the BDR was greatest in those with BPD by both spirometry and oscillometry (Table SE3).

Using published cut‐offs, we observed a bronchodilator response in 24.1% of those born ≤32 weeks gestation by spirometry compared to 7.6% of term‐born controls (*p* = .003). Oscillometry detected a bronchodilator response in 16.4% of those born preterm, compared to 4.3% of term‐born controls (*p* = .009).

Intrabreath oscillometry revealed that the magnitude of the change in inspiratory reactance (X10_insp_) and expiratory reactance (X10_exp_) following bronchodilator was greater in those born preterm (Table 3). The magnitude of this change was, however, proportional across the breath cycle, with negligible within‐breath differences in reactance (X10_insp‐exp_) (MD = −0.01, 95% CI −0.1208 to 0.101, *p* = .861) (Table 3). No significant bronchodilator‐induced decrease in inspiratory or expiratory resistance was observed in the preterm group, relative to the term‐control group (Table 3).

### 3.4 Agreement between oscillometry and spirometry outcomes

**Figure 1:**
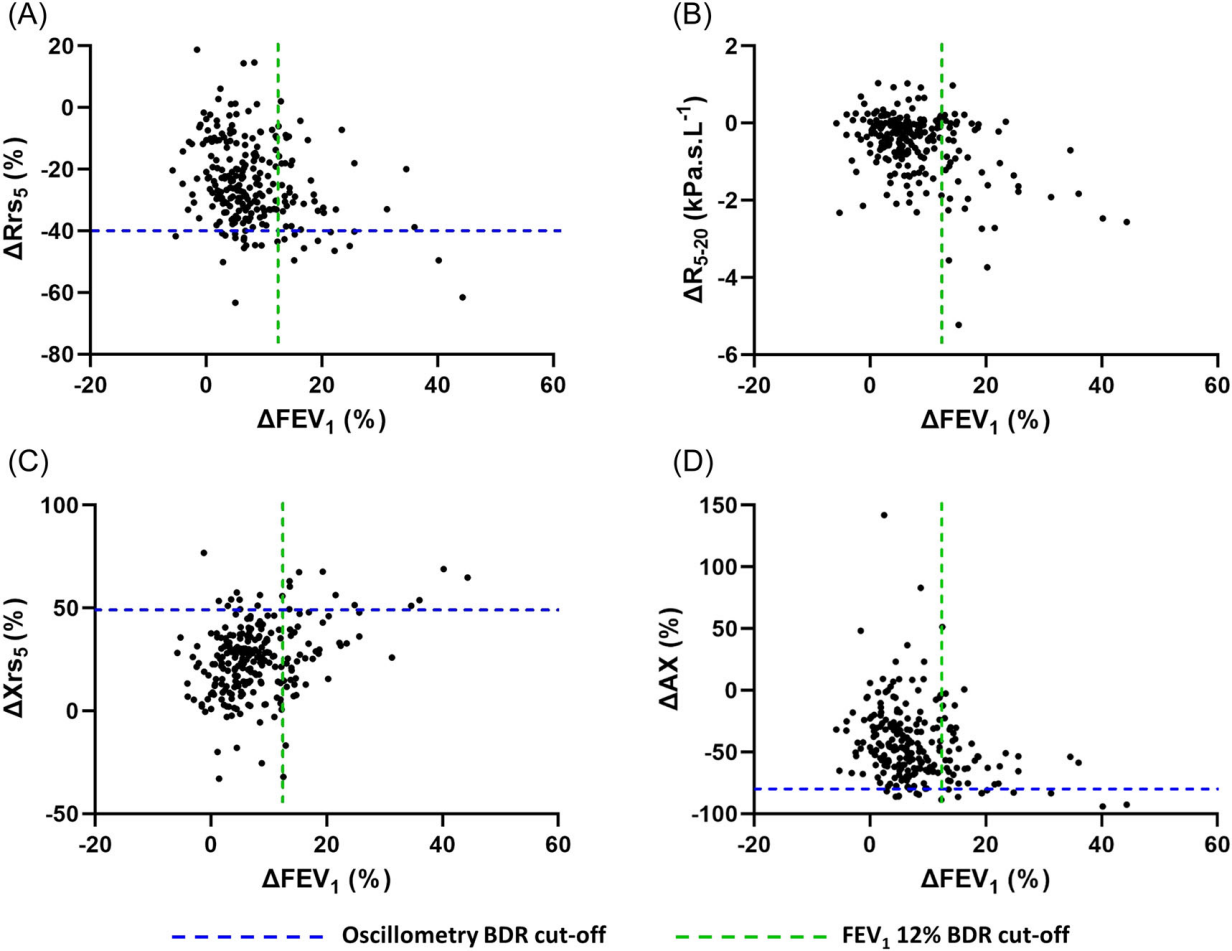
Relationship between ΔFEV_1_ and spectral oscillometry outcome measures in those born preterm. (A) ΔRrs_5_ (*R* ^2^ = .08, *p* < .0001); (B) R_5‐20_ (*R* ^2^ = .139, *p* < .0001); (C) ΔXrs_5_, % (*R* ^2^ = .1116, *p* < .0001) and (D) ΔAX (*R* ^2^ = .07, *p* < .0001). Data are presented as percent change following administration of 400 μg salbutamol via spacer (ΔFEV_1_, ΔRrs_5_, ΔXrs_5_, and AX), or absolute change (ΔR_5‐20_). AX, area under the reactance curve; FEV_1_, forced expiratory volume in 1 s; Rrs_5_, respiratory system resistance at 5 Hz; Rrs_5‐20_, difference in respiratory system resistance between 20 and 5 Hz.

Bronchodilator‐induced FEV_1_% change and change in oscillometry outcomes were correlated (Figure 1), however, these correlations were weak (*R* ^2^ < .139).

**Figure 2:**
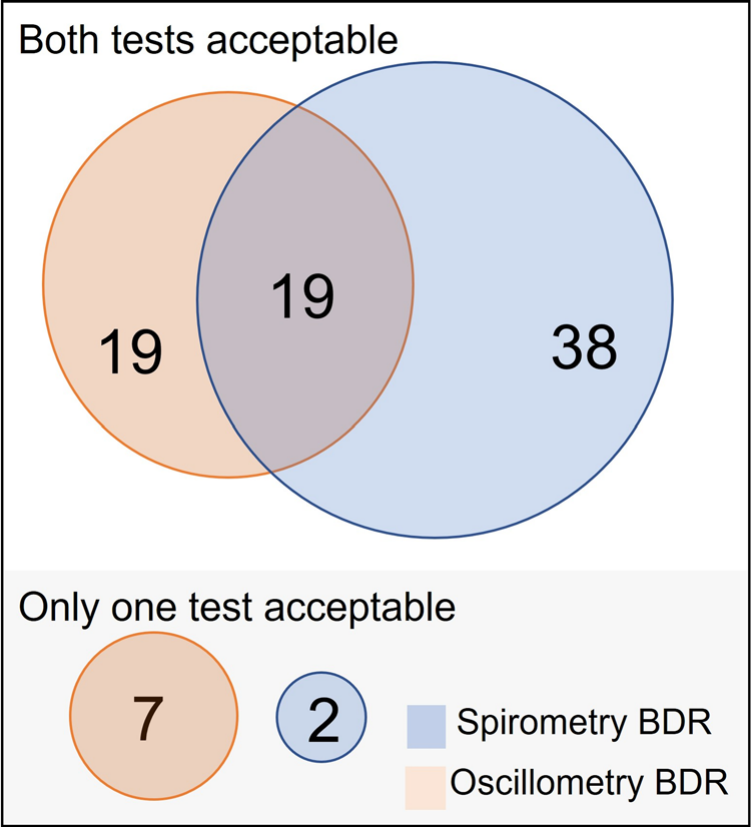
Venn diagram illustrating agreement between those with BDR via spirometry and oscillometry in those born preterm. In the preterm‐born group, of those with acceptable spirometry and oscillometry, 38 had a BDR by spirometry only, 19 by oscillometry only, and 19 had a BDR by both tests. Oscillometry identified an additional 7 participants who could not complete spirometry (*n* = 36). Spirometry identified 2 preterm participants who could not complete oscillometry measurements (*n* = 6). There was no agreement between the tests for those in the term‐born group (BDR by oscillometry *n* = 3, BDR by spirometry *n* = 5). A significant spirometry BDR was defined as ΔFEV_1_ or ΔFVC ≥ 12% (and 200 mL in for those >16 years). A significant oscillometry BDR was defined as ΔRrs_5_ ≤−40%, ΔXrs_5_ ≥ 50% or ΔAX ≤ − 80%. AX, area under the reactance curve; BDR, bronchodilator response; FEV_1_, forced expiratory volume in 1 s; FVC, forced vital capacity; Rrs_5_, respiratory system resistance at 5 Hz.

Of the 85 preterm individuals identified with a BDR (using published cut‐offs), 76 had acceptable spirometry and oscillometry. Of these individuals, only 19 (25%) showed an agreement between tests; 38 (50%) were identified by spirometry only, and 19 (25%) by oscillometry only (Figure 2). In the preterm group, agreement between tests was poor (*k* = .26; 95% CI 0.18–0.40, *p* < .001). Similarly, in the term group, the agreement was extremely poor, with no overlap between tests (*k* = −.06; 95% CI −0.10 to −0.01, *p* = .641).

Oscillometry identified an additional seven preterm‐born individuals with a BDR that could not otherwise complete acceptable spirometry. Conversely, spirometry identified two preterm individuals with a BDR and no acceptable oscillometry measures.

### 3.5 Clinical characteristics by bronchodilator response status

In the preterm group, those with a BDR by either test were more likely to have abnormal baseline lung function (Table 4). Those with a spirometry BDR had the lowest pre‐bronchodilator spirometry; this relationship was nonlinear (*R* ^2^ = .44, *p* < .001; Figure SE1). Similarly, those with an oscillometry BDR had the worst prebronchodilator oscillometry (Table 4). While a BDR was more likely in those with lower baseline lung function, spirometry was not reduced in the group with a BDR by oscillometry alone, relative to the group without a BDR (FEV_1_ z‐score MD = −0.01, 95% CI −0.66 to 0.68, *p* > .999). However, in the group that had a BDR by oscillometry but not spirometry, airway resistance was increased (Rrs_5_ z‐score MD = 0.86, 95% CI 0.07–1.65, *p* = .025) as was wheeze (33% vs. 11%, *p* = .010), compared with those without a BDR.

**Table 4:** Clinical characteristics by bronchodilator response status in those born ≤32 weeks gestation.

Variable	No BDR	Spirometry BDR	Spirometry BDR without oscillometry BDR	Oscillometry BDR	Oscillometry BDR without spirometry BDR	Both spirometry and oscillometry BDR
*N*	163	57	38	38	19	19
Male, *N* (%)	88 (54.0%)	34 (59.6%)	22 (57.9%)	20 (52.6%)	8 (42.1%)	12 (63.2%)
Caucasian, *N* (%)	139 (85.3%)	45 (78.9%)	32 (84.2%)	28 (73.7%)	15 (78.9%)	13 (68.4%)
Age, years (IQR)	13.0 (11.4–19.0)	17.6 (10.6– 19.6)	17.8 (11.0– 19.5)	12.7 (9.0– 19.2)	12.6 (9.0–18.2)	12.7 (8.1–20.3)
Height (IQR)	159.0 (143.5–170.9)	157.6 (137.9–168.2)	157.7 (142.7–169.4)	150.0 (130.4–167.5)*	148.9 (130.4–163.4)*	151.0 (127.7–168.2)
Weight (IQR)	52.8 (35.7–68.1)	50.1 (31.5–60.5)	50.5 (31.8–60.5)	46.9 (25.6–63.7)	47.7 (26.9–65.2)	46.1 (22.7–63.7)
FEV_1_ z‐score	−0.26 ± 1.04	−1.82 ± 1.02*	−1.53 ± 0.87*	−1.34 ± 1.45*	−0.27 ± 0.86	−2.40 ± 1.08*
FEV_1_/FVC z‐score	−0.71 ± 0.94	−2.17 ± 0.75*	−1.92 ± 0.71*	−1.93 ± 0.99*	−1.18 ± 0.73*	−2.67 ± 0.56*
Rrs_5_ z‐score	0.34 ± 1.21	1.24 ± 1.28*	0.90 ± 1.18*	1.56 ± 1.39*	1.20 ± 1.50*	1.92 ± 1.22*
Xrs_5_ z‐score	0.74 ± 0.87	1.74 ± 1.73*	1.18 ± 1.29*	2.07 ± 1.73*	1.29 ± 0.92*	2.84 ± 2.00*
AX z‐score	1.15 ± 1.10	2.25 ± 1.48*	1.65 ± 1.22*	2.72 ± 1.36*	1.99 ± 1.05*	3.45 ± 1.24*
Fres z‐score	1.22 ± 1.26	1.89 ± 1.46*	1.53 ± 1.28	2.42 ± 1.42*	2.03 ± 1.29*	2.94 ± 1.48*
Dr diagnosed asthma ever? *N* (%)	55/159 (34.6%)	28/57 (49.1%)	20/38 (52.6%)*	17/36 (47.2%)	9/17 (52.9%)	8/19 (42.1%)
In the past 3 months:						
Asthma medication use	16/159 (10.1%)	12/57 (21.1%)*	7/38 (18.4%)	8/37 (21.6%)	3/18 (16.7%)	5/16 (31.3%)*
Wheeze during exercise	20/158 (12.7%)	12/56 (21.4%)	8/37 (21.6%)	9/37 (24.3%)	5/18 (27.8%)	4/19 (21.1%)
Wheeze	18/158 (11.4%)	13/56 (23.2%)*	8/37 (21.6%)	11/37 (29.7%)*	6/18 (33.3%)*	5/19 (26.3%)*
Cough	72/159 (45.3%)	29/56 (51.8%)	20/37 (54.1%)	19/37 (51.4%)	10/18 (55.6%)	9/19 (47.4%)
Rattle	22/158 (13.9%)	10/56 (17.9%)	6/37 (16.2%)	7/37 (18.9%)	3/18 (16.7%)	4/19 (21.1%)
Shortness of breath	35/158 (22.2%)	19/56 (33.9%)	13/37 (35.1%)	12/37 (32.4%)	6/18 (33.3%)	6/19 (31.6%)

*Note*: Participant demographics at the time of testing are presented as mean (SD) or median (IQR) unless otherwise indicated. Data are included from participants with acceptable pre‐ and postbronchodilator spirometry and oscillometry only (*N* = 239). A significant spirometry BDR was defined as ΔFEV_1_ or ΔFVC ≥ 12% (and 200 mL in for those >16 years). A significant oscillometry BDR was defined as ΔRrs_5_ ≤−40%, ΔXrs_5_ ≥ 50%, or ΔAX ≤ − 80%. BDR, bronchodilator response. As columns do not contain discrete individuals, *T*‐test, Mann–Whitney *U*‐Test, or Chi‐sq test was used, as appropriate, to compare each column to the “No BDR” column.

Abbreviations: AX, area under the reactance curve; BDR, bronchodilator response; BPD, bronchopulmonary dysplasia; FEV_1_, forced expiratory volume in 1 s; FVC, forced vital capacity; IQR, interquartile range; Rrs_5_, respiratory system resistance at 5 Hz; SD, standard deviation.

* *p* < .05 compared with the no‐BDR group.

Baseline lung function (oscillometry and spirometry) was lowest in those with a BDR detected by both tests (e.g., FEV_1_ z‐score MD = −2.14, 95%CI −2.89 to −1.39, *p* < .001), compared to those without a BDR.

## 4 DISCUSSION

Here we describe the first comparison of bronchodilator assessments performed using oscillometry and spirometry in a preterm‐born population. Both oscillometry and spirometry demonstrate that those in the preterm group have a greater response to salbutamol, however, the magnitude of the change measured by spirometry and oscillometry was only weakly correlated. Similarly, when a response was defined as ‘significant’ using published thresholds, there was poor agreement between tests.

Spirometry is the ‘gold‐standard’ with which to assess the bronchodilator response, however, we show that oscillometry provides additional information, especially, in preterm individuals with normal spirometry and respiratory symptoms (wheeze). Spirometry may not detect mild disease that presents as ‘normal’ between exacerbations, for example, an increase in FEV_1_ ≥ 12% and 200 mL was present in only 17.3% of asthmatics in a meta‐analysis of 3 large population studies (*n* = 2833).^35^ Spirometry can remain preserved in symptomatic individuals until an advanced stage of lung disease, whilst oscillometry is sensitive to changes in small airway function^36^ and offers some advantages over spirometry in the identification of individuals with poor asthma control.^37^, ^38^ Our finding that preterm individuals with an oscillometry BDR only had increased wheeze, but normal spirometry, suggests that oscillometry has clinical value as a supplement to, rather than surrogate for−assessing the bronchodilator response in this population.

Oscillometry is not a suitable surrogate for spirometry due to poor agreement between oscillometry and spirometry‐detected BDRs, and a weak correlation between ΔFEV_1_ and oscillometry outcome measures. This has been reported previously in a retrospective review of 592 children with asthma or suspected asthma; 18% had a BDR by spirometry only, 9% by oscillometry only, and only 8% had a BDR by both tests.^39^ Oscillometry and spirometry have different measurement techniques (tidal breathing vs forced maneuvers), which likely partially explains this discrepancy. Performed during tidal breathing, oscillometry is perceived as a sensitive measure of small airway disease.^2^ In contrast, spirometry measures flow and volume during a forced maneuver, and may be better able to determine the function of larger airways.^40^ While oscillometry has been put forward as a more sensitive marker of small airways disease, it did not capture a large portion (38/57, 66.6%) of children and young adults who had a BDR by spirometry and therefore raises concerns surrounding the ability of oscillometry to detect more global changes in airway resistive forces. Notwithstanding, it may be that oscillometry has value in discriminating diseases isolated to the small airways in those born preterm.

This poor agreement between the detection of BDR by both oscillometry and spirometry is likely exacerbated by the current published definitions of a BDR using both tests. Fixed cut‐offs are typically recommended (e.g., ≥12% improvement and 200 mL in FEV_1_) and hence used here, however, the response to a bronchodilator is inversely proportional to baseline lung function and therefore also dependant on age, height, and sex.^41^ Whilst the recently published ATS/ERS guidelines have gone someway to addressing this in spirometry, recommending that the magnitude of the change should be normalized to an individual's predicted value, rather than their baseline value,^41^ this had little influence on our results (Tables SE4 and SE5). In oscillometry, there has been debate as to whether a BDR should be expressed as absolute, relative, or z‐score change, with the latest ERS technical standards advocating for relative change, until there are sufficiently robust healthy data for oscillometry to permit a z‐score approach. The published cut‐offs (ΔRrs_5_ ≤−40%, ΔXrs_5_ ≥ 50%, or ΔAX ≤ − 80%) were developed from data from healthy children^1^ and reports are emerging that these values may be too stringent for the adult population.^24^, ^42^ Using a z‐score change that incorporates the variability of the reference data set may be a suitable way to address this limitation, however, reference values for oscillometry are currently limited and, in part, device specific. There is currently no recommendation for cut‐offs for the intrabreath oscillometry measures. Nevertheless, whilst the published cut‐offs may be problematic, the weak correlation observed between ΔFEV_1_% and oscillometry outcomes supports that the poor agreement is more likely reflective of the differences in airway physiology that these tests represent, rather than purely an issue of classification.

Ours is the first study to report within‐breath changes with single‐frequency oscillometry with R10_insp‐exp_ and X10_insp‐exp_ measures pre‐ and post‐bronchodilator in preterm‐born children. Our findings suggest these within‐breath measures may be less useful than spirometry and conventional spectral oscillometry when assessing the bronchodilator response in this population. We observed no difference in the magnitude of the R10_insp‐exp_ and X10_insp‐exp_ response to a bronchodilator, rather changes were proportional across the breath cycle, and reflected global changes in resistance and reactance at 10 Hz. Recent studies have suggested that intra‐breath oscillometry measures may be more useful in detecting wheeze^43^ and predicting lower respiratory tract infections^44^ in infants and young children than spectral oscillometry, and in adults with COPD.^45^ We observed no differences in X10_insp‐exp_ measures between preterm and healthy participants at baseline, or in response to a bronchodilator, meaning that airway inhomogeneity is likely not the primary driver of airway obstruction in preterm‐born individuals. Indeed, small studies measuring ventilation inhomogeneity using multiple breath washout report no differences between preterm and term‐born infants.^46^, ^47^, ^48^ As the literature around within‐breath oscillometry is limited, there are no references for “normal” measures, and the physiology behind within‐breath outcomes remains somewhat speculative. The mechanisms underlying lung function deficits in those born preterm are likely multi‐factorial, with evidence for emphysematous change, bronchial wall thickening, and scarring,^5^ all of which may contribute to more negative respiratory reactance. Further work is needed to explore the physiology of within‐breath changes and their implications in individuals born preterm.

Consistent with previous findings, we show that oscillometry is a more feasible test than spirometry in children and young people. However, contrary to our expectations, being born preterm did not influence the feasibility of either test with similar success rates to term‐born participants.^40^ It should be noted that those with severe impairment were excluded at the time of recruitment, however, our results show that for most survivors of preterm birth, similar test success rates should be expected for those born at term in the age range studied. It should be noted that these measurements were made during a research appointment, which is not subject to the same time constraints as a clinical service, however, reviews of routine clinical testing reveal similar findings.^40^ That oscillometry (intrabreath and spectral) is feasible in a preterm‐born population reinforces its value in both a research and clinical context.

## 5 CONCLUSIONS

In the preterm population, oscillometry is a feasible and clinically useful supportive test for those unable to perform spirometry or where bronchial hyperresponsiveness is suspected in the presence of normal spirometry results. We observed poor concordance between the presence of a BDR when assessed by both spirometry and oscillometry. Given this and our finding that an oscillometry BDR (in the absence of a spirometry BDR) correlates with clinical symptoms, physiologists may wish to consider undertaking both tests in this population, noting they should not be used interchangeably. Our observations that the response to a bronchodilator as measured by oscillometry and spirometry may reflect different aspects of airway physiology warrant further investigation to advance our understanding of preterm lung disease.

## AUTHOR CONTRIBUTIONS

T. Bradshaw, E. Smith, and R. Urs wrote the original draft. T. Bradshaw and E. Smith conducted the data analysis, which was visualized and validated by S. Simpson. G. Hall, A. Wilson, and S. Simpson conceptualized the cohort studies, designed the methodology, and acquired funding. E. Smith conceptualized and designed the methodology for the comparative analysis. T. Bradshaw, R. Urs, E. Smith, D. Evans, and N. Hemy undertook project administration and investigation activities. All authors reviewed and edited the final manuscript.

## CONFLICT OF INTEREST STATEMENT

The authors declare no conflict of interest.

## Supporting information

Supporting information.

## Data Availability

Data would be made available to investigators providing a sound proposal that has been approved by an independent review committee to achieve the aims of the proposal. Proposals should be directed to shannon.simpson@telethonkids.org.au. To gain access to data, data requestors will need to sign a data access agreement.
