# Supplementary material for: Oscillometry and spirometry are not interchangeable when assessing the bronchodilator response in children and young adults born preterm
Source: Pediatr Pulmonol. 2023 Aug 4;58(11):3122–32. doi: 10.1002/ppul.26632 (PMC10947568; doi:10.1002/ppul.26632)
Supplement: Supplementary file 1 — Supporting information. [file PPUL-58-3122-s001.pdf]

**Supplementary Files**

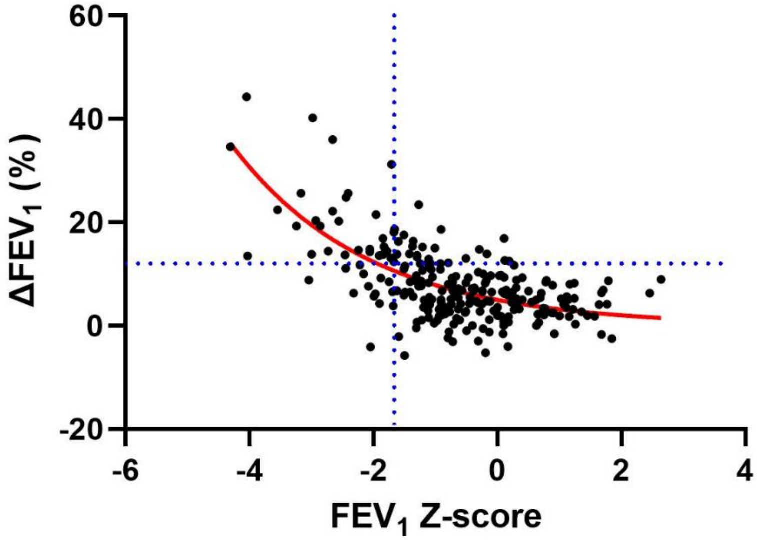


Figure E1. Relationship between pre-bronchodilator $FEV_1$ z-score and % change in $FEV_1$ in those born preterm. The dashed lines indicate the threshold for an abnormal z-score (-1.64) and the significance level for a bronchodilator response by spirometry (12% $\Delta FEV_1$). The line of best fit (red line) is non-linear ($R^2$ = 0.44, p=<0.001).

Table E1. Participant demographics and respiratory symptoms

| | Term | Preterm | p-value | BPD | No BPD |
|---|---|---|---|---|---|
| Number | 76 | 288 | | 132 | 156 |
| **Neonatal Characteristics** | | | | | |
| BPD, N (%) | | 132(45.8%) | | | |
| Gestational Age, wks | | 28.1 (26.0, 30.0) | | 25.7 (24.8, 27.0) | 29.8 (28.4, 31.0) |
| Birth Weight, z-score | | -0.06 ±0.86 | | -0.17±0.91 | 0.04±0.80 |
| Supplemental Oxygen support in NICU, days | | 8.3 (0.1, 75.0) | | 78.5 (44.0, 103.0) | 0.3 (0.0, 2.4) |
| Total Respiratory Support in NICU, days | | 18.5 (2.9, 55.0) | | 63.9 (51.0, 82.0) | 5.4 (1.2, 23.1) |
| Received Post-natal Surfactant, N (%) | | 205 (71.2%) | | 120 (92.3%) | 85 (55.6%) |
| **Participant Demographics** | | | | | |
| Age, years | 17.0 (9.6, 19.5) | 12.9 (9.9, 18.8) | 0.721 | 18.2 (10.5, 19.9) | 12.7 (9.4, 17.8) |
| Male, N (%) | 38 (50.0%) | 159 (55.2%) | 0.418 | 73 (55.3%) | 86 (55.1%) |
| Height, cm | 156.8 ±22.6 | 152.1 ±19.7 | 0.099 | 154.9±19.3 | 149.8±19.8 |
| Weight, kg | 52.4 ±22.8 | 48.6 ±21.6 | 0.195 | 50.4±20.2 | 47.0±22.7 |
| BMI | 20.1 ±4.3 | 19.9 ±4.8 | 0.716 | 20.0±4.6 | 19.8±4.9 |
| Asthma ever, N (%) | 5/73 (6.8%) | 109/279 (39.1%) | <0.001* | 57/128 | 52/151 |
| Asthma medication – past 3 months, N (%) | 2/73 (2.7%) | 40/280 (14.3%) | 0.007* | 20/129 | 20/151 |
| **In the past 3 months, when the participant did not have a cold, they have experienced:** | | | | | |
| Wheezing, N (%) | 4/73 (5.5%) | 46/277 (16.6%) | 0.016* | 24/128 | 22/149 |
| Wheeze during exercise, N (%) | 2/73 (2.7%) | 44/277 (15.9%) | 0.003* | 26/128 | 18/149 |
| Coughing, N (%) | 30/73 (41.1%) | 141/278 (50.7%) | 0.143 | 24/128 | 22/149 |
| A rattle in the chest, N (%) | 9/73 (12.3%) | 44/277 (15.9%) | 0.451 | 29/128 | 15/149 |
| Shortness of breath, N (%) | 10/73 (13.7%) | 77/277 (27.8%) | 0.013* | 41/128 | 36/149 |

Participant demographics at the time of testing are presented as n (%), mean (SD) or median (IQR). *p<0.05 compared to term-born group. BPD= bronchopulmonary dysplasia. BMI= body-mass index. Total respiratory support includes nasal continuous positive airway pressure, humidified high flow and mechanical ventilation.

Table E2. Baseline lung function in term and very preterm participants

| | Term | Preterm | p-value | BPD | No BPD |
|---|---|---|---|---|---|
| ***Spirometry*** | | | | | |
| *$FEV_1$, N valid (%)* | 70 (92.1%) | 264 (91.7%) | *0.902* | *123 (93.2%)* | *141 (90.4%)* |
| *$FEV_1$ z-score* | 0.26 ±1.12 | -0.61 ±1.21 | *<0.001** | -0.93±1.25 | -0.33±1.11 |
| *FVC, N valid (%)* | 59 (77.6%) | 217 (75.3%) | *0.679* | *105 (79.5%)* | *112 (71.8%)* |
| *FVC z-score* | 0.40 ±1.14 | 0.10 ±1.06 | *0.071* | 0.00±1.07 | 0.19±1.06 |
| *$FEV_1$/FVC z-score* | -0.21 ±0.93 | -1.06 ±1.07 | *<0.001** | -1.23±1.09 | -0.90±1.03 |
| ***Spectral Oscillometry*** | | | | | |
| *Spectral, N valid (%)* | 72 (94.7%) | 277 (96.2%) | *0.573* | *126 (95.5%)* | *151 (96.8%)* |
| *$Rrs_5$ z-score* | 0.35 ±1.31 | 0.58 ±1.29 | *0.185* | 0.81±1.25 | 0.39±1.29 |
| *$Xrs_5$ z-score* | -0.52 ±0.90 | -1.04 ±1.29 | *<0.001** | 1.23±1.43 | *0.89*±1.15 |
| *AX z-score* | 0.82 ±0.99 | 1.49 ±1.30 | *<0.001** | 1.71±1.27 | 1.30±1.30 |
| *Fres z-score* | 0.76 ±1.05 | 1.38 ±1.31 | *<0.001** | 1.69±1.41 | 1.09±1.16 |
| *$Rrs_{5-20}$ (cmH2O.s.$L^{-1}$)* | 0.28 (0.01, 0.82) | 0.73 (0.17, 1.63) | *<0.001** | *0.62 (0.11, 1.28)* | *0.87 (0.27, 1.81)* |
| ***Intrabreath Oscillometry*** | | | | | |
| *Intrabreath, N valid (%)* | 73 (98.6%) | 268 (97.5%) | *0.542* | *122 (92.4%)* | *146 (93.6%)* |
| *$R10_{insp}$ (kPa.s.$L^{-1}$)* | 4.01 (2.70, 5.07) | 4.73 (3.37,6.58) | 0.002* | *3.90 (3.03, 6.08)* | *5.22 (3.69, 7.12)* |
| *$R10_{exp}$ (kPa.s.$L^{-1}$)* | 4.43(2.97, 5.64) | 5.10 (3.48, 6.93) | 0.008* | *4.13 (3.14, 6.62)* | *5.43 (4.05, 7.39)* |
| *$R10_{insp-exp}$ (kPa.s.$L^{-1}$)* | -0.35 (-0.55, -0.12) | -0.21 (-0.53, 0.02) | 0.026* | *-0.16 (-0.39, 0.01)* | *-0.25 (-0.61, 0.02)* |
| *$X10_{insp}$ (kPa.s.$L^{-1}$)* | -0.65 (-1.22, -0.23) | -1.08 (-1.94, -0.54) | <0.001* | *-0.97 (-1.81, -0.52)* | *-1.11 (-2.02, -0.57)* |
| *$X10_{exp}$ (kPa.s.$L^{-1}$)* | -0.77 (-1.51, -0.29) | -1.32 (-2.44, -0.61) | <0.001* | *-1.03 (-2.27, -0.57)* | *-1.58 (-2.73, -0.77)* |
| *$X10_{insp-exp}$ (kPa.s.$L^{-1}$)* | 0.18 (0.00, 0.47) | 0.19 (-0.07, 0.55) | 0.561 | *0.06 (-0.10, 0.45)* | *0.28 (0.00, 0.68)* |

Data are presented as mean ±SD or median (IQR), except for the number of valid tests. *p<0·05 compared to term-born group.

$FEV_1$= forced expiratory volume in 1 s. FVC= forced vital capacity. $Rrs_5$= respiratory system resistance at 5 Hz. Fres= resonant frequency. AX= area under the reactance curve. $Xrs_5$= respiratory system reactance at 5 Hz. $Rrs_{5-20}$= difference in respiratory system resistance between 20 and 5 Hz.

Table E3. Lung function changes in response to 400mcg salbutamol in term and preterm participants

| | *Term* | *Preterm* | *p-value* | *BPD* | *No BPD* |
|---|---|---|---|---|---|
| ***Spirometry*** | | | | | |
| *$FEV_1$ Pre & Post-BD, N Valid (%)* | 66 (86.8%) | 245 (85.1%) | 0.697 | 114 (86.4%) | 131 (83.9%) |
| *$\Delta FEV_1$, L* | 0.14 (0.10, 0.18) | 0.20 (0.17, 0.22) | *0.016** | *0.24 (0.21, 0.28)* | *0.16 (0.13, 0.18)* |
| *$\Delta FEV_1$, %* | 4.4 (3.2, 5.5) | 7.8 (6.9, 8.7) | *<0.001** | *9.5 (8.0, 11.0)* | *6.3 (5.3, 7.4)* |
| *FVC Pre & Post-BD, N Valid (%)* | 57 (75.0%) | 195 (67.7%) | 0.221 | 92 (69.7%) | 103 (66.0%) |
| *ΔFVC, L* | -0.01(-0.04, 0.01) | 0.01 (-0.01, 0.02) | *0.139* | *0.02 (0.00, 0.05)* | *-0.01 (-0.02, 0.01)* |
| *ΔFVC, %* | -0.4 (-1.2, 0.4) | 0.4 (-0.1, 0.9) | *0.108* | *0.8 (0.1, 1.6)* | *-0.01 (-0.6, 0.6)* |
| ***Spectral Oscillometry*** | | | | | |
| *Pre & Post-BD, N Valid (%)* | 71 (93.4%) | 275 (95.5%) | 0.460 | 125 (94.7%) | 150 (96.2%) |
| *$\Delta Rrs_5$, %* | -19.1 (-22.6, -15.6) | -23.9 (-25.5, -22.3) | *0.013** | *-24.9 (-27.2, -22.5)* | *-23.2 (-25.4, -20.9)* |
| *$\Delta Xrs_5$, %* | 13.2 (8.0, 18.3) | 24.6 (22.3, 26.8) | *<0.001** | *25.8 (22.6, 29.1)* | *23.5 (20.4, 26.6)* |
| *ΔAX, %* | -30.0 (-37.7, -22.3) | -44.1 (-47.7, -40.6) | *0.001** | *-46.3 (-50.8, -41.7)* | *-42.4 (-47.7, -36.9)* |
| *$\Delta Rrs_{5\text{-}20}$, (kPa.s.$L^{-1}$)* | -0.14 (-0.27, -0.01) | -0.54 (-0.64, -0.43) | <0.001* | -0.5 (-0.6, -0.4) | -0.6 (-0.7, -0.4) |
| ***Intrabreath Oscillometry*** | | | | | |
| *Pre & Post-BD, N Valid (%)* | *71 (93.4%)* | *266 (92.4%)* | *0.754* | *121 (91.7%)* | *145 (92.9%)* |
| *$\Delta R10_{insp}$, (kPa.s.$L^{-1}$)* | -0.16 (-0.45, 0.12) | -0.49 (-0.71, -0.27) | *0.074* | *-0.09 (-0.37, 0.19)* | *-0.82 (-1.15, -0.49)* |
| *$\Delta R10_{exp}$, (kPa.s.$L^{-1}$)* | -0.14 (-0.42, 0.14) | -0.38 (-0.61, -0.16) | *0.175* | *-0.04 (-0.31, 0.23)* | *-0.67 (-1.01, -0.33)* |
| *$\Delta R10_{insp\text{-}exp}$, (kPa.s.$L^{-1}$)* | -0.02 (-0.16, 0.11) | -0.11 (-0.18, -0.03) | *0.305* | *-0.05 (-0.15, 0.06)* | *-0.15 (-0.26, -0.04)* |
| *$\Delta X10_{insp}$, (kPa.s.$L^{-1}$)* | 0.34 (0.24, 0.45) | 0.60 (0.48, 0.72) | 0.002* | 0.63 (0.50, 0.76) | 0.57 (0.38, 0.76) |
| *$\Delta X10_{exp}$, (kPa.s.$L^{-1}$)* | 0.22 (0.08, 0.36) | 0.58 (0.43, 0.72) | *<0.001** | *0.57 (0.40, 0.73)* | *0.58 (0.36, 0.80)* |
| *$\Delta X10_{insp\text{-}exp}$, (kPa.s.$L^{-1}$)* | 0.03 (-0.06, 0.12) | 0.02 (-0.04, 0.09) | *0.861* | *0.06 (-0.02, 0.14)* | *-0.01 (-0.11, 0.09)* |

Data is presented as mean difference (post-pre bronchodilator values) or percent change (((post-pre bronchodilator)/pre-bronchodilator)*100) (95% CI). T-Tests were used to identify differences between the term and preterm group. *p<0.05

Understanding the effect of the updated 2022 definition of a spirometry BDR for individuals born preterm

In 2022 updated guidelines on how a BDR should be defined were released by the ATS/ERS. [49] A >10% change of predicted values in $FEV_1$ or FVC was suggested to replace the well-established threshold of ≥12% and 200ml in $FEV_1$ or FVC from baseline as defined in the 2005 guidelines.[41] The updated guidelines had little impact on the interpretation of our data. Using the 2022 definition, 3/186 preterm born participants who did not previously have a BDR, were reclassified as having a BDR, while 7/59 who did have a BDR using the 2005 definition did not have a BDR using the 2022 guidelines (table E1).

| | | Spirometry $BDR^{2022}$ | | Total |
|---|---|---|---|---|
| | | No | Yes | |
| Spirometry $BDR^{2005}$ | No | 183 | 3 | 186 |
| | Yes | 7 | 52 | 59 |
| Total | | 190 | 55 | 245 |

Table E4. Agreement between the 2005 and 2022 definitions of a BDR in a preterm-born population. Using the 2022 definition, 55 participants had a BDR, while 59 had a BDR according to the 2005 definition. Agreement was excellent with a Cohen's kappa coefficient (k) of 0.89 (95% CI 0.82, 0.95, p<0.001). Spirometry $BDR^{2005}$= ≥12% and 200ml in $FEV_1$ or FVC from baseline; Spirometry $BDR^{2022}$= a >10% change of predicted values in $FEV_1$ or FVC. BDR= bronchodilator response; $FEV_1$= forced expired volumes in 1 s; FVC= forced vital capacity.

Agreement between a BDR as measured by oscillometry and spirometry (2022 guidelines) remained poor, with a Cohen's kappa coefficient (k) of 0.23 (95% CI 0.09, 0.38). This was lower than the agreement observed using the 2005 guidelines (k=0.26; 95% CI 0.18 to 0.40, p<0.001).

| | | Spirometry $BDR^{2022}$ | | Total |
|---|---|---|---|---|
| | | No | Yes | |
| Oscillometry BDR | No | 165 | 36 | 201 |
| | Yes | 21 | 17 | 38 |
| Total | | 186 | 53 | 239 |

Table E5. Agreement between the oscillometry and the 2022 definition of a BDR by spirometry in a preterm-born population. Data from individuals with acceptable spirometry and oscillometry only. Using the 2022 spirometry definition, 53 participants had a BDR, while 38 had a BDR by oscillometry. Agreement was poor with a Cohen's kappa coefficient (k) of 0.231 (95% CI 0.09, 0.38). Oscillometry BDR= a change of ≤−40% in $Rrs_5$, ≥50% in $Xrs_5$ or ≤−80% in AX. Spirometry $BDR^{2022}$= a >10% change of predicted values in $FEV_1$ or FVC. BDR= bronchodilator response; $FEV_1$= forced expired volumes in 1 s; FVC= forced vital capacity.
